# PBDE Concentrations in Women’s Serum and Fecundability

**DOI:** 10.1289/ehp.0901450

**Published:** 2010-01-26

**Authors:** Kim G. Harley, Amy R. Marks, Jonathan Chevrier, Asa Bradman, Andreas Sjödin, Brenda Eskenazi

**Affiliations:** 1 Center for Children’s Environmental Health Research, School of Public Health, University of California–Berkeley, Berkeley, California, USA; 2 Division of Laboratory Sciences, National Center for Environmental Health, Centers for Disease Control and Prevention, Atlanta, Georgia, USA

**Keywords:** fecundability, flame retardants, menstrual cycle characteristics, PBDEs, time to pregnancy

## Abstract

**Background:**

Exposure to polybrominated diphenyl ether (PBDE) flame retardants is widespread, with 97% of Americans having detectable levels. Although PBDEs have been associated with reproductive and hormonal effects in animals, no human studies have examined their association with fertility.

**Objectives:**

This study was designed to determine whether maternal concentrations of PBDEs in serum collected during pregnancy are associated with time to pregnancy and menstrual cycle characteristics.

**Methods:**

Pregnant women (*n* = 223) living in a low-income, predominantly Mexican-immigrant community in California were interviewed to determine how many months they took to become pregnant. Blood samples were collected and analyzed for PBDEs. PBDE concentrations were lipid adjusted and log_10_ transformed. Analyses were limited to PBDE congeners detected in > 75% of the population (BDEs 47, 99, 100, 153). Cox proportional hazards models modified for discrete time were used to obtain fecundability odds ratios (fORs) for the association of PBDEs and time to pregnancy.

**Results:**

We detected all four congeners in > 97% of women. Increasing levels of BDEs 47, 99, 100, 153 and the sum of these four congeners were all associated with longer time to pregnancy. We observed significantly reduced fORs for BDE-100 [adjusted fOR = 0.6; 95% confidence interval (CI), 0.4–0.9], BDE-153 (adjusted fOR = 0.5; 95% CI, 0.3–0.8), and the sum of the four congeners (adjusted fOR = 0.7; 95% CI, 0.5–1.0). PBDEs were not associated with menstrual cycle characteristics.

**Conclusions:**

We found significant decreases in fecundability associated with PBDE exposure in women. Future studies are needed to replicate and confirm this finding.

Polybrominated diphenyl ethers (PBDEs) are a class of flame retardants used in furniture, carpeting, textiles, electronics, and plastics to reduce the risk of ignition and slow down burning rates. Because PBDEs are not covalently bound to these materials, they may leach into the surrounding environment. PBDEs have been globally detected in soil (Agency for Toxic Substances and Disease Registry 2004), sediment (Hites 2004), food (Branchi et al. 2003; Hites 2004), and air (Hites 2004). Measures of PBDEs in house dust indicate widespread contamination in homes (Jones-Otazo et al. 2005; Wilford et al. 2004), and house dust may be an important human exposure pathway (Lorber 2008).

Studies indicate near universal exposure to PBDEs among the general population, with 97% of American adults sampled in the nationally representative National Health and Nutrition Examination Survey (NHANES) having detectable PBDE levels in their blood (Sjödin et al. 2008). In North America, concentrations in humans have been doubling every 4–6 years since the 1970s (Hites 2004). Levels of PBDEs are approximately 20 times higher in populations in the United States than in Europe (Hites 2004; Wilford et al. 2004; Zuurbier et al. 2006), with California residents experiencing the highest exposures (Fischer et al. 2006; Petreas et al. 2003; She et al. 2002; Zota et al. 2008), possibly due to the state’s strict flammability standards legislation.

Three technical PBDE mixtures have been commercially produced. Known as penta-, octa-, and deca-BDE, they are designated by their average degree of bromination (Alaee et al. 2003). Penta-BDE consists mainly of PBDE congeners with four to six bromines (e.g., BDE-47 and BDE-99), octa-BDE contains congeners with 6–10 bromines (including BDE-153), and deca-BDE consists almost exclusively of BDE-209 (La Guardia et al. 2006). Although penta- and octa-BDE have been banned for use in the United States, exposure continues because these mixtures are present in furniture and other home products manufactured before 2004. Deca-BDE continues to be used, primarily in electronic products. The congeners found in the greatest concentration in human serum in the U.S. general population are BDEs 47, 99, 100, and 153, which are the primary components of penta-BDE (Sjödin et al. 2008). However, few studies have been able to measure BDE-209 in serum.

In animal studies, PBDEs have been shown to affect neurobehavior (Branchi et al. 2002; Dufault et al. 2005; Eriksson et al. 2001, 2002; Johansson et al. 2008; Viberg et al. 2003a, 2003b, 2004b, 2006) and to alter sex hormone and thyroid hormone homeostasis (Ellis-Hutchings et al. 2006; Fowles et al. 1994; Hallgren and Darnerud 2002; Hallgren et al. 2001; Stoker et al. 2004; Zhou et al. 2001, 2002), but few studies have investigated potential health effects in humans. Thyroid hormones play a strong role in the regulation of ovulation, menstrual cycle regularity, and fertility (Krassas 2000; Poppe and Velkeniers 2004). PBDEs, which exhibit estrogenic and antiestrogenic properties, have also been associated with delayed onset of puberty (Lilienthal et al. 2006; Stoker et al. 2004) and altered circulating levels of estradiol (Talsness et al. 2008) in female animals.

Only one small human study (*n* = 20) has examined the association of PBDEs and female reproductive function. Chao et al.(2007) reported that higher PBDE levels in breast milk of new mothers were associated with shorter prepregnancy menstrual cycle length (< 30 days), but this finding was not statistically significant when models were adjusted for maternal age, body mass index, and parity. Our study of pregnant women in California is the first to examine the association of PBDE concentration in blood with time to pregnancy in humans.

## Methods

### Study participants

Participants were pregnant women enrolled in the Center for the Health Assessment of Mothers and Children of Salinas (CHAMACOS) study, a longitudinal birth cohort study of environmental exposures and reproductive health in the Salinas Valley, an agricultural region of California. Pregnant women were recruited from six prenatal care clinics serving a low-income, predominantly Mexican-immigrant population. Women were eligible to participate if they were < 20 weeks gestation at enrollment, spoke English or Spanish, were eligible for low-income health insurance (Medicaid), and were ≥ 18 years of age. All women provided written informed consent, and study procedures were approved by the Committee for the Protection of Human Subjects at the University of California–Berkeley.

A total of 601 pregnant women enrolled in the study, and of these, 343 women had PBDEs measured in serum collected during pregnancy. (The main reason for missing PBDE measurement was insufficient volume of serum remaining after other, higher priority toxicants were measured.) Women with PBDE measurements did not differ significantly from those without PBDE measurements on any of the main demographic or fertility-related variables listed in Table 1 (data not shown). The PBDE results for 24 women were excluded because of problems with the automated equipment used to process samples in that batch. We also excluded one woman who was missing time-to-pregnancy data, one woman using fertility medication during the month of conception, and 94 women using contraception at the time of conception (contraceptive failures), leaving a final sample of 223 women who contributed 984 cycles to the analysis. In subsequent sensitivity analyses, the 94 contraceptive users were included in the sample, increasing the sample size to 317 women and 1,120 cycles.

### Data collection

In-person interviews were conducted at the time of enrollment [median, 13 weeks gestation; interquartile range (IQR), 10–18 weeks]. The beginning of the pregnancy was determined by asking the woman the date of her last menstrual period (LMP) or, if she did not know (4%), by using the LMP estimate from the clinical ultrasound. Women were then asked, “How many months did it take to become pregnant? In other words, for how many months had you been having sexual intercourse without doing anything to prevent pregnancy?” A calendar was used to help with recall. The interval between stopping contraception and becoming pregnant was considered the “time-to-pregnancy period” and was marked on the calendar to aid recall about exposures during that time period.

Women were also asked whether they had had regular menstrual periods in the year before the pregnancy and, if so, how many days were in her cycle. Additional questions asked about hormonal contraceptive use in the year before the pregnancy, frequency of intercourse, use of fertility medication, use of contraception (either regularly or inconsistently) during the month of conception, and whether she had been actively trying to get pregnant.

Information was also gathered about potential confounders, including maternal and paternal age, education, country of birth, and years of residence in the United States. Women were asked about their reproductive history, including prior pregnancies and breast-feeding history. Histories of gynecologic conditions, urogenital surgeries, or sexually transmitted infections were combined into a single variable indicating a history of relevant medical conditions. Body mass index was calculated from the mother’s measured height and self-reported prepregnancy weight. Information was also gathered on other exposures that might affect fertility, including smoking and alcohol and caffeine consumption in the time before conception. Because this is an agricultural population, we also gathered information on maternal and paternal work in agriculture, pesticide use in the home, and residence near agricultural fields during the time-to-pregnancy period.

### PBDE exposure

Maternal blood was collected near the end of the second trimester of pregnancy (median, 26 weeks gestation; IQR, 25–27 weeks). PBDE concentrations in maternal serum were determined using gas chromatography/isotope-dilution high-resolution mass spectrometry (Sjödin et al. 2004). Serum was analyzed for 10 PBDE congeners: BDEs 17, 28, 47, 66, 85, 99, 100, 153, 154, and 183. Quality assurance and control procedures included the use of blanks and spiked samples in each sample batch (Sjödin et al. 2004). Maternal serum was analyzed for total cholesterol and triglyceride levels using standard enzymatic methods (Roche Chemicals, Indianapolis, IN) (Phillips et al. 1989). PBDE concentrations were reported both as wet weight (picograms per unit serum) and lipid adjusted (nanograms per gram of lipids). The limits of detection (LODs) for PBDE analyses were between 0.2 and 0.7 ng/g lipids for all congeners, except for BDE-47, which ranged from 0.8 to 2.6 ng/g lipids.

### Statistical analysis

PBDE concentrations below the LOD but for which a signal was detected were coded with the concentration obtained. Data below the LOD for which no signal was detected were coded as the lowest concentration obtained for that congener divided by the square root of 2 (Hornung and Reed 1990). Statistical analysis was limited to congeners that were detected in > 75% of the population (BDEs 47, 99, 100, and 153). The detection frequencies for these target analytes were high, ranging from 99.6% (BDE-47) to 97.3% (BDE-100). A sum PBDE variable was created by adding the four highly detected congeners (both as nanograms per gram of lipids and on the molar scale); the variable summed by weight is presented unless otherwise noted.

Cox proportional hazards models modified for discrete time data were used to estimate fecundability odds ratios (fORs) for the association between PBDE congeners and time to pregnancy (Baird 1988). The fOR estimates the odds of achieving pregnancy in any given month, conditional on not having become pregnant in a previous cycle. Thus, fORs < 1 indicate longer time to pregnancy and decreased fecundability. The unit of analysis was months, with pregnancies occurring in the first cycle coded as 1 month. Time to pregnancy was censored at 13 months to lessen the influence of outliers and because couples receiving fertility treatment generally seek it after attempting to conceive for > 12 months. Separate models were run for each PBDE congener. Statistical analyses were performed using STATA 10 (StataCorp, College Station, TX).

Covariates were included in the adjusted Cox models if they were independently associated with both time to pregnancy and the summed PBDE variable or if their exclusion from the model changed the coefficient for the sum of PBDEs by ≥ 10%. Table 1 lists maternal factors tested as covariates; paternal age, smoking, and work in agriculture were also considered as covariates. Covariates retained in the final models were maternal age and indicator variables for years of residence in the United States, history of gynecologic conditions, hormonal contraceptive use in the year before conception, breast-feeding in the 2 months before conception, and caffeine consumption in the 3 months before pregnancy. Covariates were categorized as shown in Table 1 unless otherwise specified. We have previously shown that markers of maternal pesticide exposure are associated with longer time to pregnancy (Harley et al. 2008); thus, three variables to assess pesticide exposure during the prepregnancy period were also retained in the final model: work in agriculture, home pesticide use, and residence within 200 feet of an agricultural field. Because menstrual cycle irregularity and history of gynecologic conditions might be on the causal pathway, final models were tested with and without these variables. Frequency of intercourse was tested as a covariate, but its inclusion did not change the results.

After final models were identified, sensitivity analyses were conducted to determine whether the relation of PBDEs and fecundability changed when *a*) the population was limited to women actively trying to become pregnant (*n* = 107) or to primiparous women (*n* = 92), *b*) the population was expanded to include women who were using contraception at the time of pregnancy (i.e., contraceptive failures) (*n* = 317), or *c*) time to pregnancy was censored at 7, 10, or 15 months rather than 13 months (Joffe et al. 2005). Results of these models were compared with the main models to assess consistency of results under these differing conditions. Additionally, because using lipid-adjusted exposure measures can result in bias (Schisterman et al. 2005), we also ran all models using wet-weight PBDE concentrations (picograms per gram of serum) and including lipids as a covariate in the model. Because results were similar regardless of how lipids were considered, results using the lipid-adjusted PBDE values (nanograms per gram of lipids) are reported.

Because altered menstrual cycle function could be a mechanism by which PBDEs affect fecundability, we also examined the association of maternal PBDE levels with menstrual cycle characteristics. The association of PBDE levels with irregular menstrual cycles was examined using logistic regression. Among women with regular menstrual cycles, we examined the association of PBDE levels with cycle length as a continuous variable using linear regression. This analysis was limited to women who reported that they had not used hormonal contraception in the previous year (*n* = 191), because this could affect menstrual cycle characteristics. *p*-Values for significance < 0.05.

## Results

Participants in the study were mostly young, Mexican-immigrant women (Table 1). Educational attainment was low, with 78% of participants having less than a high school education. Most participants lived near agricultural fields, and almost half worked in agriculture before pregnancy. Fewer than half of the women were actively trying to get pregnant at the time of conception; an additional 37% were not concerned about whether they became pregnant, and 15% were trying not to become pregnant. Median time to pregnancy was 3 months (range, 1–180 months). Fifteen percent of participants took longer than 12 months to conceive.

Factors associated with longer time to pregnancy in this population included irregular menstrual cycle, breast-feeding within 2 months of conception, and living in the United States for ≥ 11 years (compared with recent immigrants living in the United States for ≤ 5 years). Women working in agriculture, living within 200 feet of a field, or using pesticides in their homes also had longer time to pregnancy. Women who were using hormonal contraception in the year before pregnancy had shorter time to pregnancy.

Table 2 shows PBDE levels in maternal blood during pregnancy. More than 97% of women had detectable levels of BDEs 47, 99, 100, and 153, and these four congeners were highly correlated (*r* = 0.77–0.95). These high detection frequencies were similar to those observed for Mexican-American women participating in the 2003–2004 NHANES survey (Sjödin et al. 2008). Analyses for NHANES were conducted by the same laboratory and had similar LODs as in this study. Geometric mean concentrations of BDEs 47, 99, 100, and 153 in our sample were slightly lower than for 18- to 40-year-old Mexican-American women in NHANES, possibly because a higher percentage of women in our sample than in the NHANES population were recent immigrants to the United States. The strongest predictor of PBDE levels in our population was years of residence in the United States, with higher PBDE concentrations seen among women who had lived in the United States longer (Bradman et al. 2008). We detected all 10 congeners analyzed in this population of pregnant Californians; specifically, we detected BDE-17 in 1%, BDE-28 in 46%, BDE-66 in 15%, BDE-85 in 45%, BDE-154 in 40%, and BDE-183 in 31% of women. Because we detected these congeners in < 75% of women, we did not include them in the time-to-pregnancy analysis.

We ran separate Cox models for each congener. Table 3 shows the unadjusted and adjusted fORs associated with increasing concentrations of the four dominant PBDE congeners, individually and summed. After controlling for confounders, each 10-fold increase in the concentration of BDE-100 and BDE-153 was associated with a 40% decrease [fOR = 0.6; 95% confidence interval (CI), 0.4–0.9; *p* < 0.01] and 50% decrease (fOR = 0.5; 95% CI, 0.3–0.8; *p* < 0.01), respectively, in the odds of achieving pregnancy in each month. BDEs 47 and 99 were also associated with 20–30% lower odds of pregnancy, but these findings were of borderline statistical significance (*p* = 0.07 and *p* = 0.11, respectively). When concentrations of all four congeners were summed, a 10-fold increase in the sum PBDEs was associated with 30% decreased odds of pregnancy each month (fOR = 0.7; 95% CI, 0.5–1.0; *p* = 0.04).

When the population was limited to women actively trying to become pregnant (*n* = 107), all four PBDE congeners were associated with reduced fecundability at *p* < 0.05, and the magnitude of the effect was greater than in the entire population (Table 3).

**Figure 1:**
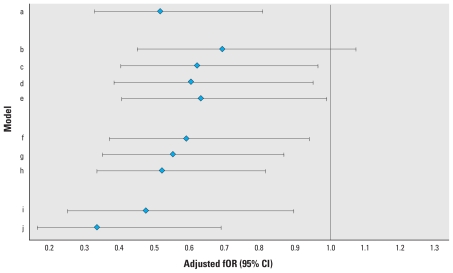
Adjusted fOR and 95% CIs for association of BDE-153 in maternal serum with time to pregnancy, according to various sensitivity analyses. (Time to pregnancy is censored at 13 months unless otherwise specified.) Models: a, main population (*n* = 223); b, main plus all pregnancies resulting from contraceptive failure (time to pregnancy = 0; *n* = 317); c, main plus contraceptive failure with irregular contraceptive use (time to pregnancy = duration of irregular use/2; *n* = 249); d, main plus contraceptive failure with regular contraception use (time to pregnancy = 0; *n* = 291); e, main plus all contraceptive failure (time to pregnancy = 0 or duration/2; *n* = 317); f, main, censored at 7 months (*n* = 223); g, main, censored at 10 months (*n* = 223); h, main, censored at 15 months (*n* = 223); i, limited to primiparas (*n* = 92); j, limited to those actively trying for pregnancy (*n* = 107).

In additional sensitivity analyses (i.e., limiting the population to primiparas, expanding the population to include women experiencing contraceptive failure, or changing the right censoring of the time-to-pregnancy variable), results were similar to those reported in Table 3. We found statistically significant reductions in fORs for BDE-100 and BDE-153 in almost all of the sensitivity analyses, whereas the fORs for BDE-47 and BDE-99 were reduced but of borderline statistical significance. Figure 1 shows sensitivity analyses for BDE-153 [for the other three congeners, see Supplemental Material, Figure 1 (doi:10.1289/ehp.0901450)]. Results were also similar when PBDE congeners were summed on a molar basis and when PBDEs were expressed on a wet-weight basis (data not shown). We found no interaction of pesticide exposure and PBDEs.

Irregular menstrual cycles did not appear to be the mechanism by which PBDEs were affecting fertility. PBDE concentrations were not associated with irregular menstrual cycles in this population. Among women with regular cycles, higher PBDE levels were associated with shorter cycle length, but this finding was not statistically significant [see Supplemental Material, Table 1 (doi:10.1289/ehp.0901450)]. When we included irregular menstrual cycles as a covariate in the models, the association of PBDEs and time to pregnancy remained similar and was actually slightly stronger than when we did not control for this variable, suggesting that irregular menstrual cycles was not on the causal pathway.

## Discussion

Similar to other studies, we found that the PBDE congeners present in the highest concentration were BDEs 47, 99, 100, and 153. We detected these congeners, components of the penta-BDE mixture, in > 95% of women. We found that increased concentrations of PBDEs in serum were associated with longer time to pregnancy in a population of low-income, pregnant Latinas in California. All four congeners, as well as the sum of the four congeners, were associated with reduced fecundability.

This study provides the first evidence that PBDEs may affect human fertility. Infertility affects an estimated 2.1 million couples in the United States (Chandra et al. 2002), causing considerable emotional strain and often resulting in medical intervention with substantial financial costs to prospective parents and society. Because exposure to PBDEs is ubiquitous in industrialized nations, even small decreases in fecundability may have wide-reaching public health impacts. Although penta- and octa-BDE are no longer in widespread use, and legislation to restrict the use of deca-BDE is being considered in many countries and states, exposure to PBDEs is likely to continue for many years as existing furniture, carpet padding, electronics, and other consumer products containing these flame retardant chemicals age and degrade. Analysis of house dust from homes of low-income children in our study region has found the highest reported levels of PBDEs to date (Quiros L, unpublished observations), suggesting that the next generation of Californians may be more extensively exposed.

The mechanisms by which PBDEs might affect fecundability are unclear, although one possibility is through disruptions of the hypothalamic–pituitary–thyroid axis. Several animal studies have shown that PBDE exposure is associated with reduced free and total thyroxine (T_4_) (Darnerud et al. 2007; Fernie et al. 2005; Hallgren et al. 2001; Kuriyama et al. 2007; Lema et al. 2008; Zhou et al. 2001, 2002). *In utero* PBDE exposure in female rats has been associated with reproductive effects, including decreased ovarian weight and follicle number (Lilienthal et al. 2006; Talsness et al. 2008), that are similar to effects seen with induced hypothyroidism (Dijkstra et al. 1996).

However, although animal studies suggest a link between PBDEs and hypothyroidism, PBDE exposure is associated with subclinical hyperthyroidism in this population (Chevrier J, Harley KG, Bradman A, Gharbi M, Sjodin A, Eskenazi B, unpublished observations) and with increased T_4_ or decreased thyroid-stimulating hormone in other epidemiologic studies (Hagmar et al. 2001; Meeker et al. 2009; Turyk et al. 2008). Both high and low thyroid hormone levels can disturb normal menstrual patterns, and treatment to restore thyroid hormone homeostasis often improves fertility (Cramer et al. 2003; Poppe and Velkeniers 2004).

A second possible mechanism by which PBDE exposure may be affecting fertility is via the hypothalamus–pituitary–gonadal axis. In *in vitro* estrogen receptor binding assays, lower brominated PBDEs, including BDEs 28, 47, and 100, exhibit estrogenic activity, whereas higher brominated compounds, such as BDE-153 and BDE-190, show antiestrogenic properties (Hamers et al. 2006; Meerts et al. 2001). Antiandrogenic activity and progesterone receptor antagonist activity has been observed for some congeners (Hamers et al. 2006). In animal studies, prenatal exposure to PBDEs has been associated with delayed puberty in females (Lilienthal et al. 2006; Stoker et al. 2004) and higher fetal resorption rates (Talsness et al. 2005).

The findings of this study are limited to the four main congeners, BDEs 47, 99, 100, and 153, that we detected in almost all women. The levels of these four congeners were highly correlated (*r* = 0.77–0.95), restricting our ability to distinguish whether one particular congener is exerting a stronger effect. Additionally, because of analytical constraints, we were unable to measure BDE-209.

An additional limitation of this study is that we measured PBDE levels only in women and not in their partners. Although we examined paternal factors such as age, smoking, and agricultural work, we were unable to examine the role of male PBDE exposure in this analysis.

We conducted this study in a farmworker community, and findings may not be generalizable to other populations. We previously found that potential exposure to pesticides, as assessed by occupational and residential patterns, was associated with reduced fecundability in this population (Harley et al. 2008). However, all models controlled for these variables, and current pesticide exposure was not associated with PBDE levels. Further, we did not find any associations of time to pregnancy with maternal concentrations of persistent organochlorine pesticides, such as dichlorodiphenyltrichloroethane (DDT), or polychlorinated biphenyls (PCBs).

Time-to-pregnancy studies may be prospective, following a group of women trying to become pregnant, or retrospective, like the present study. By design, this study was limited to women who were already pregnant; thus, infertile and subfertile women were underrepresented in the sample. However, if PBDEs are associated with decreased fecundability, including only the most fecund women likely would underestimate the effect, biasing the findings toward the null.

An advantage of our study design was that it included both women who were trying to conceive and those who were not. Approximately 30% of all live births in the United States are unplanned (Finer and Henshaw 2006). Thus, prospective studies that are limited to women who are trying to conceive may underrepresent the most fertile women and may overrepresent highly motivated couples, making them susceptible to other selection biases.

An additional advantage of this study is that, although it was retrospective, interviews occurred near the beginning of the pregnancy, ensuring a short recall time for time-to-pregnancy information. A recent validation study found that women exhibit poor recall of time to pregnancy (Cooney et al. 2009), although the recall period of 10 years in that study was much longer than for our study. Validation studies have also demonstrated poor self-report of menstrual cycle characteristics (Jukic et al. 2008; Small et al. 2007), which may affect the interpretation of these results. We measured PBDE concentrations during pregnancy. Because these PBDE congeners are persistent, concentrations in blood collected during pregnancy likely are a good representation of levels during the time-to-pregnancy period.

This study is the first to report that higher PBDE concentrations in women’s blood are associated with significantly longer time to pregnancy, and this finding needs to be replicated in other populations. The study population of predominantly Mexican immigrants living in an agricultural community is a distinctive group, and findings may not be generalizable to all women. The relationship among PBDE exposure, length of time in the United States, and fertility is somewhat complex. Although we controlled for both time in the United States and pesticide exposure, this study should be replicated in a population that is not subject to these factors. If confirmed, this finding would have strong implications to women trying to conceive given that exposure to PBDEs is nearly universal in the United States and many other countries.

## Figures and Tables

**Table 1:** Selected characteristics of study population and their association with time to pregnancy: CHAMACOS study, 1999–2000 (*n* = 223).

Characteristic	*n* (%)	Time to pregnancy [months, median (IQR)]
Maternal age^a^ (years), all participants [median (IQR)]	23.9 (21.5–27.3)	3 (1–7)

Race/ethnicity		
Non-Hispanic white	5 (2.2)	4 (4–15)
Hispanic	214 (96.0)	3 (1–7)
Other	4 (1.8)	2 (1–21)

Years of residence in the USA		
≤ 5	138 (61.9)	2 (1–6)
6–10	39 (17.5)	3 (1–9)
≥ 11	25 (11.2)	4 (2–15)*
Entire life	21 (9.4)	4 (1–7)

Education		
≤ 6th grade	88 (39.5)	3 (1–6)
7–12 grade	85 (38.1)	3 (1–6)
Completed high school	50 (22.4)	4 (1–9)

Prior pregnancy		
No	92 (41.3)	3 (1–7)
Yes	131 (58.7)	3 (1–7)

Trying to get pregnant		
No	116 (52.0)	3 (1–6.5)
Yes	107 (48.0)	3 (1–7)

More than 12 months to become pregnant		
No	189 (84.8)	2 (1–4)
Yes	36 (15.3)	25 (15–39)*

Menstrual cycles		
Regular	175 (78.5)	2 (1–6)
Irregular	48 (21.5)	5 (2–14)*

Hormonal contraceptive use in year before pregnancy		
No	149 (66.8)	3 (1–9)*
Yes	74 (33.2)	3 (1–5)

Breast-fed in 2 months before pregnancy		
No	213 (95.5)	3 (1–6)
Yes	10 (4.5)	9 (4–43)*

History of gynecologic conditions		
No	178 (79.8)	3 (1–6)
Yes	45 (20.2)	4 (1–9)

Smoked^b^		
No	199 (89.6)	3 (1–6)
Yes	23 (10.4)	4 (1–9)

Caffeine consumption^b^		
No	30 (13.5)	1.5 (1–5)
Yes	193 (86.6)	3 (1–7)

Mother worked in agriculture^b^		
No	121 (54.3)	3 (1–6)
Yes	102 (45.7)	3 (1–8)*

Lived ≤ 200 feet of agricultural field^b^		
No	187 (83.9)	3 (1–6)
Yes	36 (16.1)	4 (1–16.5)*

Pesticides applied in home^b^		
No	202 (90.6)	3 (1–6)
Yes	21 (9.4)	5 (3–12)*

a Age at beginning of time-to-pregnancy period.

b During the time-to-pregnancy period.

* Associated with longer time to pregnancy in univariate proportional hazards models (*p* < 0.05).

**Table 2:** Lipid-adjusted PBDE concentrations (ng/g lipid) in serum of pregnant women: CHAMACOS study, 1999–2000 (*n* = 223).

PBDE congener	Percent detect	Geometric mean (95% CI)	IQR
BDE-47	99.6	14.9 (12.9–17.2)	7.4–25.2
BDE-99	99.6	4.4 (3.9–5.1)	2.2–64
BDE-100	97.3	2.8 (2.4–3.2)	1.5–4.0
BDE-153	97.8	2.5 (2.2–2.8)	1.2–3.6

**Table 3:** Association of log_10_-transformed maternal PBDE concentration (ng/g lipid) with time to pregnancy: CHAMACOS study, 1999–2000.

PBDE congener^a^	fOR (95% CI)	
	Crude	Adjusted^b^
All women (*n* = 223)		
BDE-47	0.69 (0.48–1.01)	0.73 (0.51–1.04)
BDE-99	0.74 (0.51–1.06)	0.76 (0.54–1.06)
BDE-100	0.59 (0.39–0.87)*	0.61 (0.42–0.89)*
BDE-153	0.53 (0.34–0.82)*	0.52 (0.33–0.81)*
Sum PBDE	0.66 (0.44–0.97)*	0.68 (0.47–0.98)*

Women trying to become pregnant (*n* = 107)		
BDE-47	0.60 (0.37–0.97)*	0.51 (0.31–0.84)*
BDE-99	0.61 (0.38–0.98)*	0.58 (0.37–0.89)*
BDE-100	0.54 (0.32–0.90)*	0.42 (0.23–0.78)*
BDE-153	0.54 (0.31–0.93)*	0.34 (0.16–0.69)*
Sum PBDE	0.56 (0.34–0.94)*	0.45 (0.25–0.78)*

a Separate models for each congener.

b Adjusted for mother’s age, years of residence in the Unites States, history of gynecologic conditions, hormonal contraceptive use in previous year, breast-feeding in previous 2 months, caffeine consumption, and pesticide exposure in before pregnancy (work in agriculture, home pesticide use, and residence within 200 feet of an agricultural field).

* *p* < 0.05.
